# Single‐cell RNA sequencing reveals transcriptional profiles of monocytes in HBV‐infected pregnant women during mid‐pregnancy

**DOI:** 10.1111/jcmm.17746

**Published:** 2023-04-20

**Authors:** Hongyan Wang, Xia Li, Kaiyue Yang, Fanfan Guo, Xiaona Wang, Ziyan Zhao, Yanju Jia, Fan Gao, Guiqin Bai

**Affiliations:** ^1^ Department of Obstetrics and Gynecology the First Affiliated Hospital of Xi'an Jiaotong University Xi'an China; ^2^ Clinical Research Center the First Affiliated Hospital of Xi'an Jiaotong University Xi'an China; ^3^ Gene Joint Laboratory the First Affiliated Hospital of Xi'an Jiaotong University Xi'an China

**Keywords:** difference, HBV‐infected pregnant women, monocytes, single‐cell RNA sequencing

## Abstract

There is a growing body of evidence that innate immunity also plays an important role in the progression of hepatitis B virus (HBV) infection. However, there is less study on systematically elucidating the characteristics of innate immunity in HBV‐infected pregnant women. We compared the features of peripheral blood mononuclear cells in three healthy pregnant women and three HBV‐infected pregnant women by single‐cell RNA sequencing. 10 DEGs were detected between groups and monocytes were the main expression source of most of the DEGs, which involved in the inflammatory response, apoptosis and immune regulation. Meanwhile, qPCR and ELISA were performed to verify above genes. Monocytes displayed immune response defect, reflecting poor ability of response to IFN. In addition, eight clusters were identified in monocytes. We identified molecular drivers in monocytes subpopulations.*TNFSF10+* monocytes, *MT1G+* monocytes and *TUBB1+* monocytes were featured with different gene expression pattern and biological function.*TNFSF10+* monocytes and *MT1G+* monocytes were characterized by high levels of inflammation response.*TNFSF10+* monocytes, *MT1G+* monocytes and *TUBB1+* monocytes showed decreased response to IFN. Our results dissects alterations in monocytes related to the immune response of HBV‐infected pregnant women and provides a rich resource for fully understanding immunopathogenesis and developing effective preventing HBV intrauterine infection strategies.

## 1 INTRODUCTION

Chronic hepatitis B (CHB) is an infectious disease that threatens human health worldwide. Nowadays, about 3 billion people have been infected with hepatitis B virus (HBV) worldwide, including about 350–400 million chronic HBV carriers.^1^ Mother‐to‐child transmission is the main route of HBV transmission, accounting for about 30%–50% of patients with CHB, with improvements in detection methods and the standardisation of blood transfusion procedures.^2^, ^3^ Although a prevention program involving hepatitis B vaccination and hepatitis B immunoglobulin can block transmission substantially, about 5%–10% of newborns are still infected with HBV, possibly via intrauterine infection.^4^, ^5^

The progression of HBV infection is closely associated with the immune response.^6^ There is increasing evidence that innate immunity also plays an important role in the progression of HBV infection.^6^, ^7^ Therefore, it is very important to study innate immunity in HBV‐infected pregnant women to guide the development of therapies aimed at blocking intrauterine transmission. Monocytes are an important part of innate immunity. After HBV infection, activated monocytes produce and secrete a large number of inflammatory cytokines and chemokines to recruit inflammatory cells and clear target cells in an active and synergistic manner. Antigens are presented to T cells by MHC II molecules, resulting in the activation and proliferation of T cells, triggering an antiviral response.^6^, ^8^ During chronic HBV infection, cytokines play a critical role in immune regulation and inflammation. They inhibit viral replication and influence the persistence of HBV infection. Interferons α, β and γ have essential roles in the innate immune response against CHB. Chronic HBV infection suppresses the production of IFN and further reduces cellular responses to IFN, affecting the activation of other cellular pathways and mechanisms.^6^, ^9^, ^10^

However, few studies have evaluated the functional association between HBV infection in pregnant women and monocytes. In addition, activated monocytes cannot be effectively used as therapeutic targets, mainly due to their substantial heterogeneity and complex functions, requiring the accurate isolation of monocyte subsets and localisation of activated or dysfunctional subsets.^11^ With the development of single‐cell RNA sequencing technology, it has become possible to accurately isolate different monocyte subpopulations and reveal their functional differences.^12^, ^13^, ^14^

To evaluate differences in immune function between HBV‐infected pregnant women and healthy pregnant women and to guide the development of therapies blocking mother‐to‐child transmission, we used single‐cell RNA sequencing to obtain the transcriptional profiles of peripheral blood mononuclear cells (PBMCs) from three HBV‐infected pregnant women and three healthy pregnant women, with a focus on alterations in monocytes. Our results showed that innate immune response was weakened in HBV‐infected pregnant women. A functional enrichment analysis revealed monocyte subclusters that played a role in HBV‐infected pregnant women. These findings improve our understanding of the dynamics of monocyte subsets in HBV‐infected pregnant women and provide therapeutic targets for reducing mother‐to‐child transmission of HBV.

## 2 MATERIALS AND METHODS

### 2.1 Human subjects

Peripheral blood samples were collected from pregnant women in their second trimester who were admitted to the Department of Obstetrics and Gynaecology of the First Affiliated Hospital of Xi'an Jiaotong University from August 2019 to September 2019, including three pregnant women with hepatitis B and three healthy pregnant women. The pregnant women with hepatitis B met the following criteria: (1) positive HBsAg, HBeAg, HBcAb and HBV DNA > 1.0 × 10^7^ IU/mL; (2) ALT and AST were normal; (3) gestational age was 22–23 weeks; and (4) antiviral therapy was not administered before blood collection. Healthy pregnant women met the following criteria: (1) negative HBsAg, HBeAg, HBeAb and HBcAb; (2) ALT and AST were normal; (3) without other diseases, such as hepatitis C, syphilis and AIDS. The gestational age was 24–25 weeks. Clinical and virological parameters are described in Table S1. This study was approved by the Ethics Committee of the First Affiliated Hospital of Xi'an Jiaotong University (Approval number: No. 201985). Informed consent was obtained from all participants.

### 2.2 Isolation of peripheral blood mononuclear cells

Five millilitres of peripheral blood from all subjects were collected into EDTA anticoagulated tubes. PBMCs were separated using Ficoll‐Paque PREMIUM 1.084 g/L sterile solution (GE Healthcare, Uppsala, Sweden) by density gradient centrifugation. Then, 90% FBS + 10% DMSO was added to the cryopreservation tube, followed by storage at −80°C in a refrigerator.

### 2.3 Single‐cell RNA sequencing

Single‐cell RNA‐seq libraries were prepared using Chromium Single Cell 30 Reagent v2 Kits according to the manufacturer's protocol. PBMC suspensions were loaded on the Chromium Single Cell Controller Instrument (10× Genomics) to generate single‐cell gel beads in emulsions (GEMs). About 15,000–20,000 cells were added to each channel with a targeted cell recovery of 10,000 cells. Captured cells were lysed, and the released RNAs were barcoded by reverse transcription in individual GEMs. Then, these libraries were sequenced on the Illumina sequencing platform (HiSeq X Ten), and 150 bp paired‐end reads were generated.

### 2.4 Single‐cell RNA‐seq data preprocessing

The Cell Ranger pipeline (version 3.1.0) provided by 10× Genomics was used to demultiplex cellular barcodes, map reads to the genome and transcriptome using the STAR aligner and down‐sample reads as required to generate normalized aggregate data across samples, producing a matrix of gene counts versus cells. The unique molecular identifier (UMI) count matrix was obtained using the R package Seurat^15^ (version 3.0). To remove low‐quality cells and likely multiplet captures, which are a major concern in microdroplet‐based experiments, we filtered out cells with UMI/gene numbers >±2 times the standard deviation of the mean, assuming a Gaussian distribution of values. Following the visual inspection of the distribution of cells by the fraction of mitochondrial genes expressed, we further discarded low‐quality cells in which a certain percentage of counts belonged to mitochondrial genes. Library size normalisation was performed using Seurat with the filtered matrix to obtain the normalized counts. The most highly variable genes across single cells were identified using the method described in Macosko et al.^16^ Briefly, the average expression and dispersion were calculated for each gene, and genes were placed into several bins based on expression. A principal component analysis (PCA) was performed to reduce the dimensionality of the log‐transformed gene‐barcode matrices of highly variable genes. Cells were clustered based on a graph‐based clustering approach and were visualized in two‐dimensional space using tSNE. The likelihood ratio to simultaneously evaluate changes in mean expression and in the percentage of cells with expression was used to identify significant DEGs between clusters. Here, the R package SingleR,^17^ a novel computational method for unbiased cell type recognition based on scRNA‐seq data, was used to independently infer the cell of origin of single cells and identify cell types.

### 2.5 Differentially expressed genes

DEGs were identified using the Seurat package.^18^ *p* < 0.05 and |log2fold change| > 1.5 (or |log2fold change| < 0.67) were set as the thresholds for significant differential expression. GO and KEGG pathway enrichment analyses of DEGs were performed using R based on the hypergeometric distribution.

### 2.6 GO analysis and KEGG pathway enrichment analysis

The Cluster profiler^19^ of R Package was used to analyse the biological functional genes of the top 100 DEGs with the largest expression in each cluster or cell type, and the biological functional gene set was the focus of the study. Pathways with significant enrichment were selected according to *p* value, and the significant value of *p* was 0.05.

### 2.7 Gene set variation analysis

GSVA (Version 1.30.0)^20^ was used for differential pathway analysis. To evaluate the activity of gene sets among clusters, the average expression and log2fold change of gene sets for each cluster were first determined. Then the LIMMA package (Version 3.38.3) was used to compare the pathway activity between the two groups. *p* < 0.05 was set as the threshold for significance.

### 2.8 Cell culture

Monocytes, human monocytic THP‐1 cells, were purchased from the American Type Culture Collection (ATCC, Manassas, VA, USA) and grown in RPMI‐1640 culture medium (Gibco, Life Technologies, Grand Island, NY, USA) supplemented with 10% foetal bovine serum (Gibco, Life Technologies), 50 U/mL penicillin and 50 μg/mL streptomycin (P/S; Gibco, Invitrogen) at 37°C with 5% CO_2_.

### 2.9 Cell stimulation

Monocytes were plated in 12‐well plates (Costar, Corning Incorporated, Corning, NY, USA) at 5 × 10^5^ cells/well, unless indicated otherwise. Cells were stimulated with normal maternal serum and hepatitis B maternal serum for 48 h at 37°C. Cells were harvested for RNA isolation and qPCR.

### 2.10 qPCR

Total mRNA was isolated from cells using the RNA Fast 200 Kit (Fastagen, Shanghai, China) according to the manufacturer's instructions. cDNA was synthesized with PrimeScript RT Master Mix (TaKaRa, Shiga, Japan) following the manufacturer's protocol. PCR assays were performed using a CFX Connect Real‐Time System (Bio‐Rad, Hercules, CA, USA) and SYBR Green (Genstar, Beijing, China). The primers are shown in Table S2. The reaction volume was set to 20 μL and conditions were as follows: 95°C for 10 min, followed by 40 cycles of 15 s at 95°C, 30 s at 60°C and 30 s at 72°C. Data were analysed by the 2^−ΔΔCt^ method, and relative mRNA expression levels are reported as fold differences.

### 2.11 Enzyme‐linked immunosorbent assay

IL‐1β concentrations in the cell culture supernatant and maternal serum were measured using commercial enzyme‐linked immunosorbent assay (ELISA) kits for IL‐1β (Hengyuan, Shanghai, China; HS1973‐Hu), according to the manufacturer's instructions. The lower limit of detection was 1 pg/mL.

### 2.12 Statistical analysis

Statistical analyses were performed using GraphPad Prism 8.0 (La Jolla, CA, USA). Data are shown as means ± standard error of the mean. Unpaired Student's *t*‐tests were used to compare means between groups. For all analyses, *p* < 0.05 was considered significant. (ns: no significance, *p < 0.05).

## 3 RESULTS

### 3.1 Single cell atlas of PBMCs in HBV‐infected pregnant women and healthy pregnant women

**FIGURE 1:**
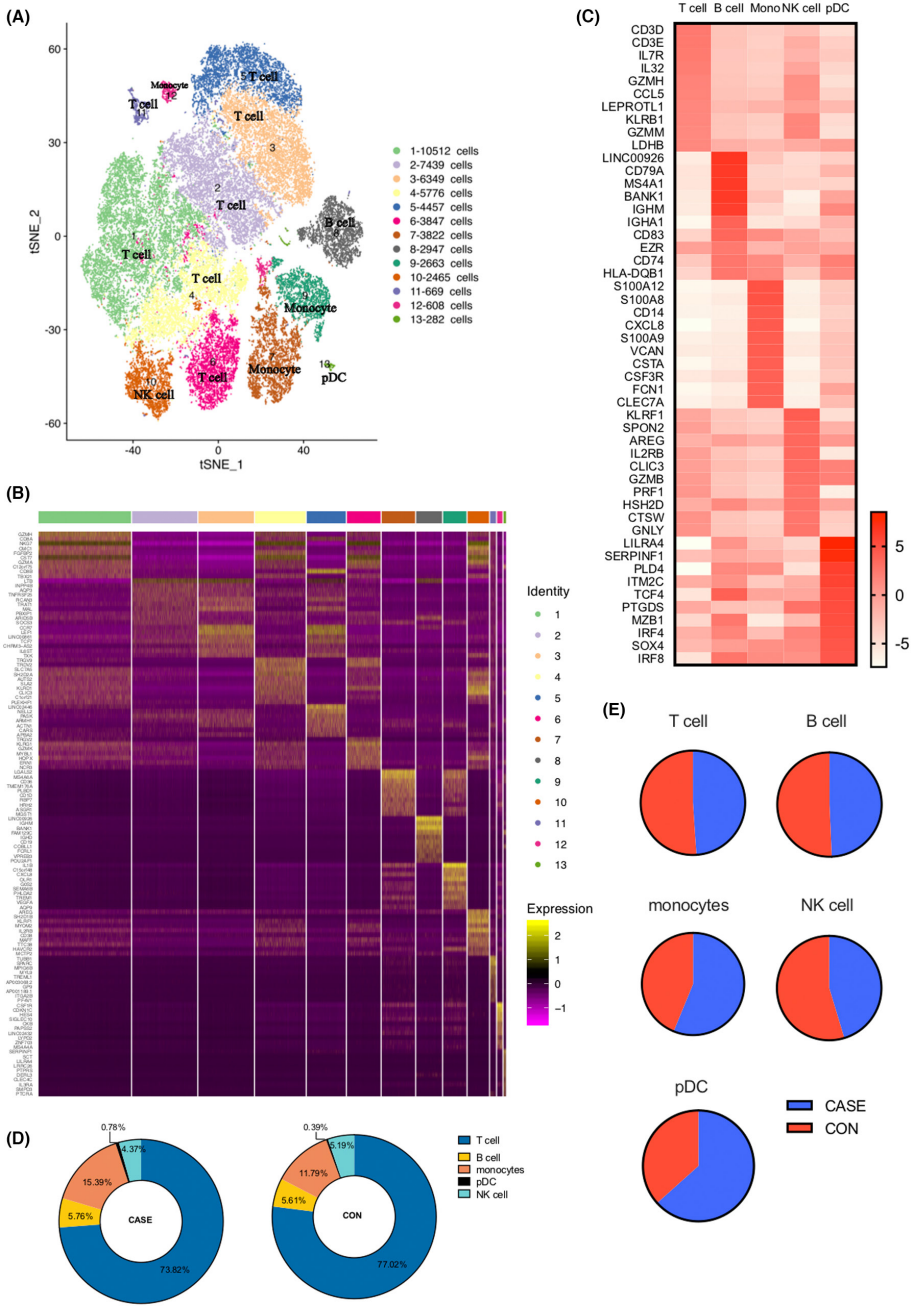
Overview of scRNA sequencing of PBMCs. (A) t‐SNE visualisation of 51,836 single cells, showing 13 clusters and 5 cell types identified in PBMCs. Each dot corresponds to a single cell and is coloured according to the cell cluster. (B) Heatmap showing the expression of marker genes for 13 cell clusters. Colours represent the average log normalized gene expression. Markers were ordered to visualize the differences between cell clusters. (C) Heatmap showing the canonical marker genes for each cell type. Markers were ordered to visualize the differences between cell types. (D, E) Pie charts showing the proportion of the five cell types in the PBMCs of CASE and CON groups. Percentages were obtained by dividing the number of cells in each cell type by the total number of cells in all patients or in all healthy controls.

We collected fresh PBMCs derived from three HBV‐infected pregnant women (CASE group) and three healthy (CON group) pregnant women according to the inclusion and exclusion criteria. The gestational week of the HBV‐infected pregnant women was between 22 and 23 weeks, and all of them were at the stage of HBeAg‐positive chronic infection. No antiviral therapy were used before pregnancy and before blood sampling in this study (Table S1). After single‐cell RNA sequencing and aggregating all sample data using Cell Ranger,^15^, ^21^ we finally obtained 51,836 cells, including 27,390 cells from HBV‐infected pregnant women and 24,446 cells from healthy pregnant women. According to the expression of canonical marker genes,^22^, ^23^, ^24^ we identified 13 clusters and 5 cell types by the unbiased *t*‐distributed neighbourhood embedding algorithm (t‐SNE), including T cells (*CD3D, CD3E, CD3G*), B cells (*CD79A, CD19, MS4A1*), monocytes (*CD14, CD16, S100A12*), NK cells (*KLRF1, GNLY*) and plasmacytoid dendritic cells (pDc, *LILRA4*) (Figure 1A). We showed heat maps of the top 10 specifically expressed genes for each cluster and five cell types (Figure 1B,C). By further comparison, we observed that there was no significant difference in the proportions of these five cell types between the two groups, even though monocytes and pDc slightly increased in case group (Figure 1D,E).

### 3.2 Differentially expressed genes in PBMCs were enriched in monocytes

**FIGURE 2:**
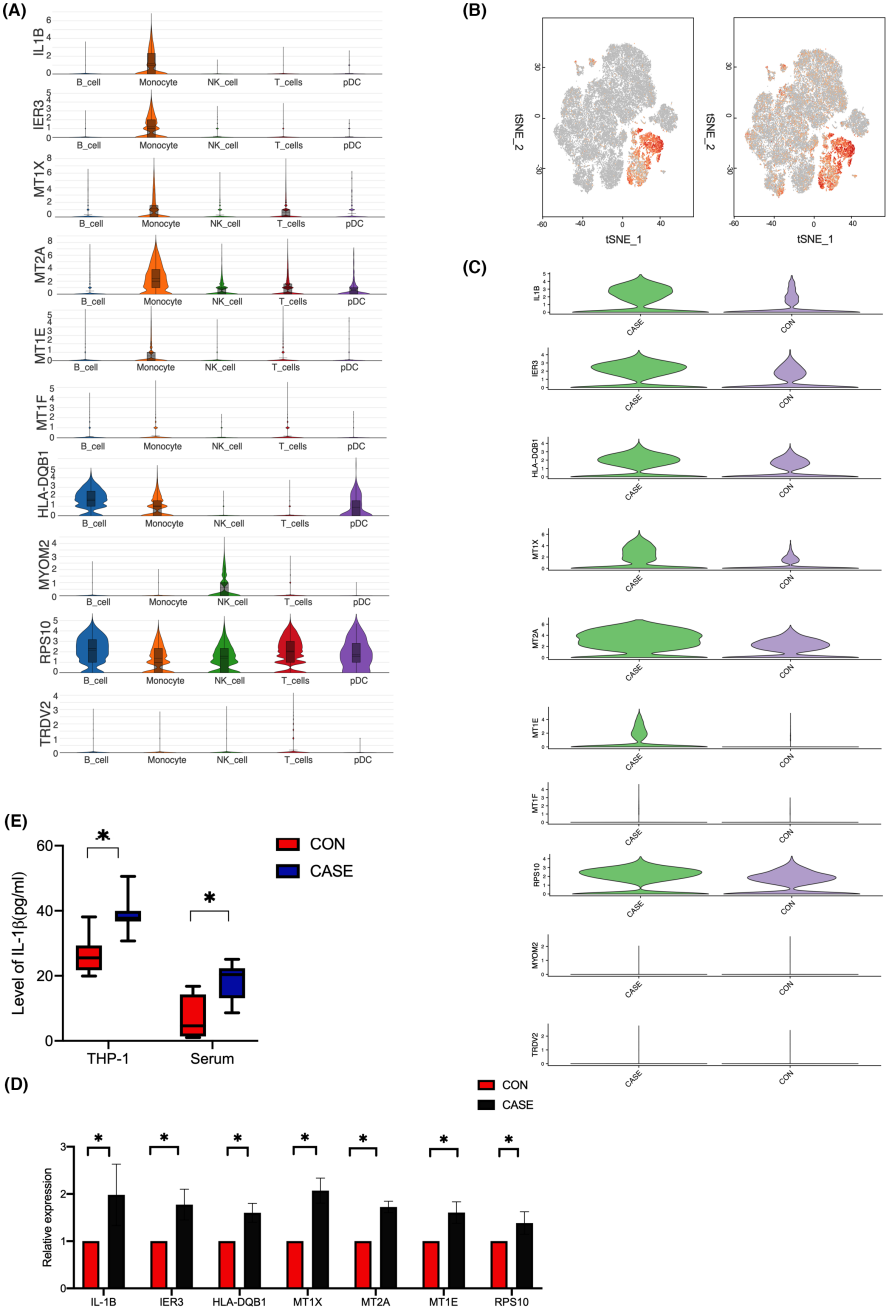
Differentially expressed genes in PBMCs. (A) Violin plots displaying levels of differentially expressed genes in five cell types. The *y*‐axis shows relative expression levels of transcripts in single cells. (B) Feature plots showing that monocytes were enriched for IL1B (left) and IER3 (right). (C) Violin plots displaying levels of differentially expressed genes in monocytes between CASE and CON groups. The *y*‐axis shows relative expression levels of transcripts in single cells. (D) Representative DEGs in serum stimulated THP‐1 by a qPCR analysis (*n* = 6). CASE versus CON: *p < 0.05. (E) ELISA of IL‐1β levels. The left dot plots represent the levels in the cell supernatant of THP‐1 (*n* = 3). The right dot plots represent the levels in serum of healthy and HBV‐infected pregnant women (*n* = 2 each). *p < 0.05.

To explore the differences between different groups, we used DEGs method to analyse the enrichment of PBMC differential genes in HBV‐infected pregnant women and healthy pregnant women. The results showed that there were 10 differentially expressed genes (|log2fc| > 1.5 and *P*adj < 0.05) between two groups. Compared to healthy pregnant women, *MT1X, MT2A, MT1E, MT1F, RPS10, IL1B, IER3* and *HLA‐DQB1* were upregulated in HBV‐infected pregnant women. *MYOM2* and *TRDV2* were downregulated. To figure out which cell type was most responsible for expression of these differentially expressed genes, we analysed the mRNA levels of 10 DEGs in five cell populations and found that *IL1B* and *IER3* were solely enriched in monocytes, with low or no expression in other cell types. The expression of levels of *MT1X*, *MT2A* and *MT1E* were relatively high in monocytes. *MYOM2* was only highly expressed in NK cells. *HLA‐DQB1* was more highly expressed in B cells, monocytes and plasmacytoid dendritic cells than in other cell types, and *RPS10* was expressed in all cell types. *MT1F* and *TRDV2* showed low expression in all cell types (Figure 2A). Two plots of tSNE showed the distribution of *IL1B* and *IER3* in PBMCs (Figure 2B).

Our study suggested that the differentially expressed genes of PBMC between HBV‐infected pregnant women and healthy pregnant women were mainly concentrated in monocytes. Therefore, we speculated that monocytes were important cells leading to immune changes in HBV‐infected pregnant women and played an important role in intrauterine HBV infection. Hence, we specially focused on alteration of monocytes in subsequent analysis.

The expression levels of 10 genes were compared in monocytes of HBV‐infected and healthy pregnant women (Figure 2C). *IL1B, IER3, MT1X, MT2A, MT1E, RPS10* and *HLA‐DQB1* were all upregulated in monocytes of HBV‐infected pregnant women. The remaining three genes were low expression.

To verify results of the single‐cell RNA sequencing, we stimulated the human monocyte cell line THP‐1 with serum from healthy pregnant women and HBV‐infected pregnant women in the middle phase of pregnancy to simulate immune microenvironment of human HBV infection, respectively. Total RNA was extracted from THP‐1 and qPCR was performed to verify mRNA levels of *IL1B, IER3, MT1X, MT2A, MT1E, RPS10* and *HLA‐DQB1* in monocytes of the two groups. The results were consistent with those obtained by single‐cell RNA sequencing (Figure 2D).

IL‐1β, a protein encoded by IL1B, is a proinflammatory factor secreted by monocytes, which is involved in inflammatory response against HBV. We detected IL‐1β protein levels in the cell culture supernatant. Quantitative analysis showed a significant increasing expression in the case group compared with the control group (*p* < 0.05). Furthermore, levels of IL‐1β in serum was also detected in two groups. IL‐1β levels were significantly higher in HBV‐infected women than in healthy pregnant women (*p* < 0.05; Figure 2E).

### 3.3 Functional defects in monocytes of HBV‐infected pregnant women

**FIGURE 3:**
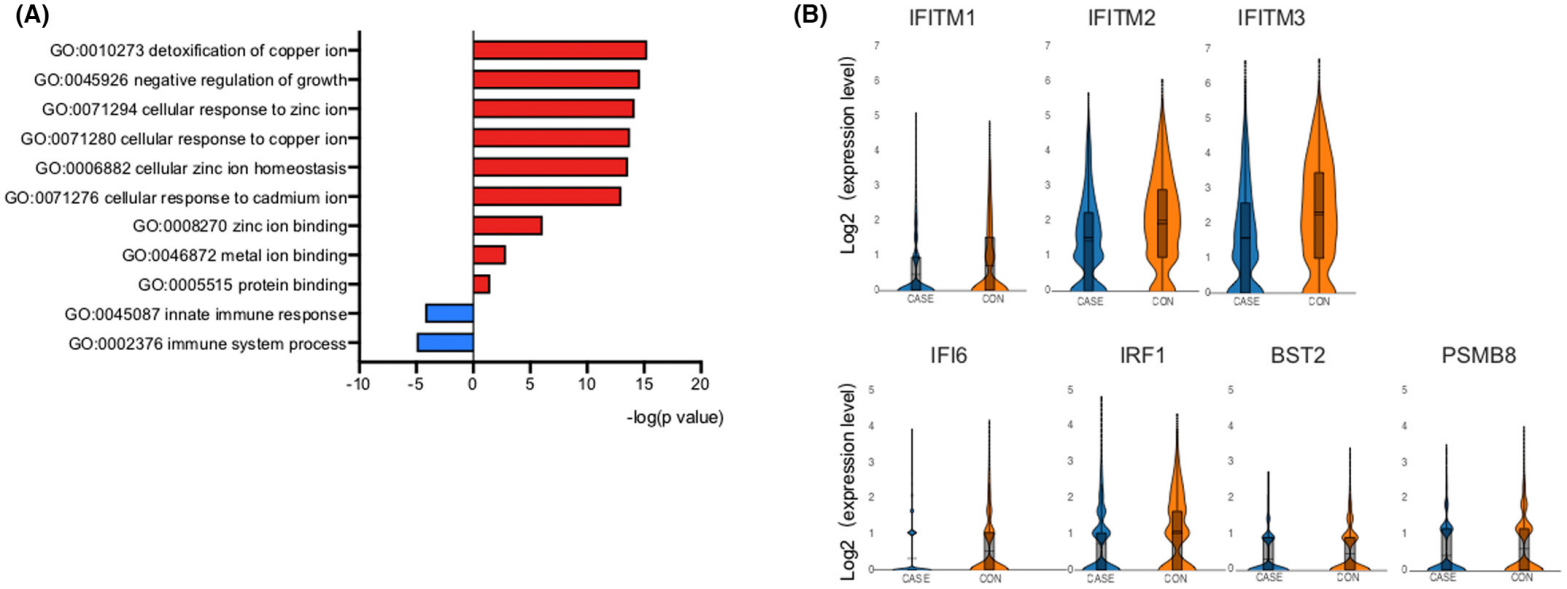
Functional defects in monocytes of HBV‐infected pregnant women. (A) GO enrichment analysis of DEGs between monocytes in the CASE and CON groups. (B) Violin plots showing the expression of interferon‐stimulated genes between CASE and CON groups. The *y*‐axis shows relative expression levels in single cells.

Since there was not differences of the cell frequency of monocytes between groups, we further analysed the transcriptional alteration in monocytes. A differential expression analysis and GO analysis of monocytes between HBV‐infected pregnant women and healthy pregnant women were performed and only nine DEGs were identified. Compared to control group, the upregulated DEGs in monocytes were enriched in biological processes in case group, such as the negative regulation of growth, cellular zinc ion homeostasis and immune regulation. More importantly, functions related to the immune system and innate immune response were downregulated (Figure 3A). It is necessary to investigate which aspects function of monocytes existed changes.

Next, we compared the expression levels of antiviral interferon‐stimulated genes^13^ in monocytes.*IFITM1, IFITM2, IFITM3, IFI6, IRF1, BST2* and *PSMB8* were clearly lower than those in monocytes in HBV‐infected pregnant women, suggesting that the antiviral activity of monocytes from HBV‐infected pregnant women was weakened (Figure 3B).

Our results indicated that, monoctyes of HBV‐infected pregnant women showed enhanced ability of immune regulation, whereas the immune response was defective, reflecting in the response to interferon and to virus defence, which may be involved in the mechanism of HBV intrauterine infection.

### 3.4 Heterogeneity of monocyte clusters

**FIGURE 4:**
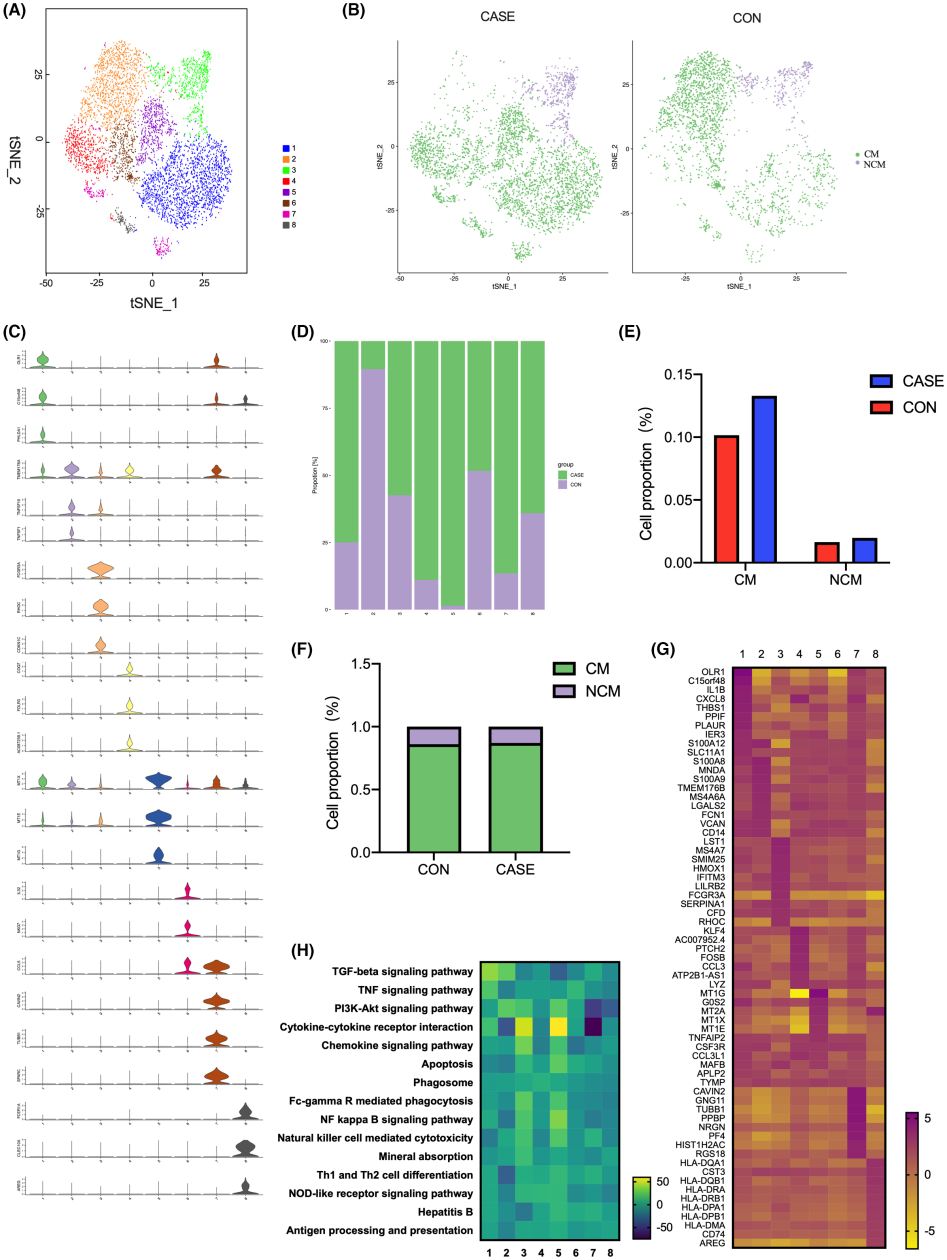
Overview of monocyte subclusters. (A) t‐SNE visualisation of monocytes, showing eight clusters. Each dot corresponds to a single cell and is coloured according to the cell cluster. (B) tSNE visualisation of monocytes coloured according to the two major monocyte subtypes, classical monocytes (CM) and non‐classical monocytes (NCM). (C) Violin plots showing the expression of marker genes for eight cell clusters. (D) Bar plots showing the expression of marker genes for eight cell clusters. (E) Bar plots showing the proportion of CM and NCM in PBMCs between CASE and CON groups. (F) Bar plots showing the proportion of CM and NCM in monocytes between CASE group and CON group. (G) Heatmap showing the top 10 highly expressed genes for the 8 clusters. (H) Heatmap showing the top GSVA pathways for the 8 clusters.

However, it was difficult to deeply understand the changes of innate immunity in HBV‐infected pregnant women at the overall level of monocytes, which might be related to the heterogeneity of monocytes. Next, we performed dimensionality reduction clustering analysis on monocytes to identify specific transcriptional signatures. We identified eight clusters and found differences in the distribution of clusters between HBV‐infected pregnant women and healthy pregnant women (Figure 4A–C). The proportion of monocytes assigned to cluster 2 in HBV‐infected pregnant women was significantly lower than that in healthy pregnant women, the proportion of cells assigned to cluster 6 was slightly lower and the proportion of cells assigned to other clusters all were higher (with the most significant increase in cluster 5) (Figure 4D).

According to the expression of canonical markers of monocyte subtypes,^22^ two monocyte subpopulations were identified: classical monocytes (CM, clusters 1, 2, 4, 5, 6, 7 and 8) and non‐classical monocytes (NCM, cluster 3) (Figure 4B). Compared to healthy pregnant women, the proportions of CM and NCM did not change in HBV‐infected pregnant women. Moreover, there also were no significant differences in the proportions of CM and NCM between the two groups, revealing that there was no transformation between monocyte subtypes (Figure 4E,F).

To gain further insight into the transcriptional signature of the monocyte subclusters, we determined top 10 genes highly expressed in each cluster of monocytes, and the gene expression patterns of eight clusters showed significant heterogeneity (Figure 4G). Cluster 1 (*OLR1+*) showed inflammatory activation with expression signatures of proinflammatory cytokine/chemokine genes, such as *IL1B* and *CXCL8*, and apoptosis factors (*IER3* and *PPIF*). Cluster 2 (*TNFSF10+*) expressed high levels of proinflammatory genes, such as S100 genes, *IL1B*, *VCAN* and *LYZ* with a high proinflammatory ability and *FCN1* (which inhibits viral entry into host cells). Cluster 3 (*FCGR3A+*) was enriched for *IFITM3* and *FCGR3A*, related to the interferon response, inhibition of viral replication and ability to kill target cells after viral infection. Cluster 4 (*COQ7+*) was characterized by decreased expression of metallothionein genes and the enrichment of genes related to regulatory transcription (*KLF4* and *FOSB*). Cluster 5 (*MT1G+*) was characterized by high expression levels of metallothionein genes (*MT1G, MT2A, MT1X* and *MT1E*) and inflammation‐related genes, such as *CCL3L1, TNFAIP2* and *CSF3R*, exhibiting a high proinflammatory feature and immune regulation capacity. Cluster 6(*IL32+*) had a similar gene expression profile to that of cluster 2 (*TNFSF10+*). Cluster 7 (*TUBB1+*) showed high expression levels of *PPBP* and *PF4*, related to chemotaxis and neutrophil degranulation. Cluster 8 (*CLEC10A+*) expressed high levels of class II HLA molecules, suggesting capacities of antigen presentation in this clusters.

In addition, a one‐to‐many GSVA was then performed to reveal the characteristics of pathways enriched in immune responses in each cluster (Figure 4H). As for cytokine–cytokine receptor signalling pathway, Cluster 5 showed significant enrichment, whereas cluster 2 and cluster 7 showed lower expression.

Therefore, we further focused on these three clusters to excavate the alterations of monocytes of HBV‐infected pregnant women.

### 3.5 Functional changes of monocyte subsets in HBV‐infected pregnant women

To estimate biologic processes to dysregulated genes in monocyte subsets, we evaluated DEGs between HBV‐infected and healthy pregnant women within each specific subpopulation.

Volcano maps were generated for the visualisation of DEGs in individual monocyte clusters of HBV‐infected pregnant women compared with those of healthy pregnant women. Almost every monocyte cluster, except for cluster 6, was characterized by high expression of metallothionein genes in HBV‐infected pregnant women and the upregulation of the mineral absorption pathway (Figure S1). Metallothionein participates in immune regulation by regulating zinc homeostasis and the oxidative stress response, suggesting that immune regulation is enhanced in monocytes of HBV‐infected pregnant women. We focused on clusters 2, 5 and 7, since clusters 2 and 7 showed enrichment for the inflammatory response and all three clusters showed the down‐regulation of antiviral response‐related pathways in case group.

**FIGURE 5:**
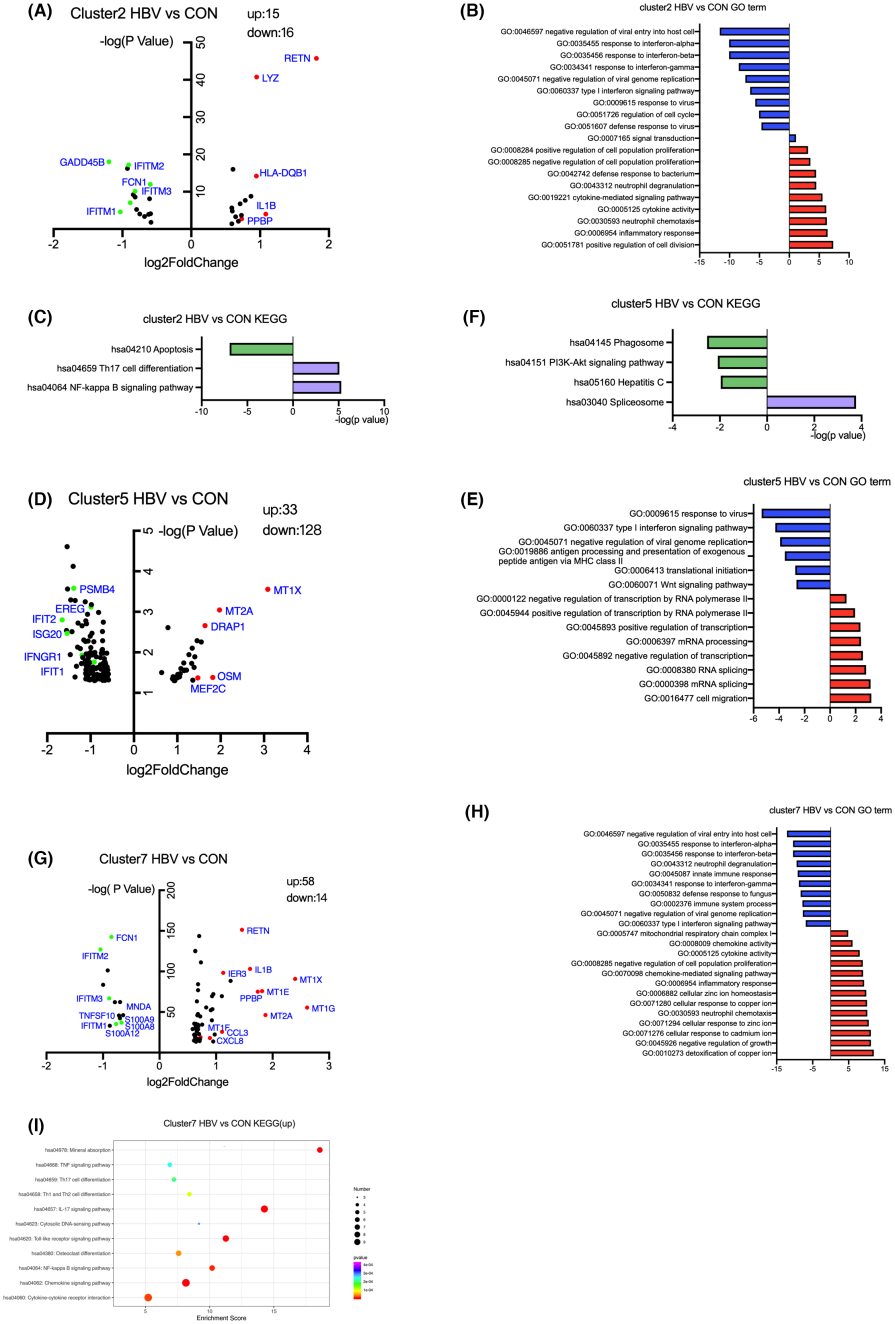
DEGs in monocyte subclusters 2, 5 and 7 and functional enrichment analyses. (A) Volcano plot showing the differentially expressed genes in cluster 2 between HBV‐infected pregnant women and healthy pregnant women. (B) GO enrichment analysis of DEGs in cluster 2 between CASE and CON. (C) KEGG enrichment analysis of cluster 2. (D) Volcano plot showing DEGs in cluster 5. (E) GO enrichment analysis of DEGs in cluster 5. (F) KEGG enrichment analysis of cluster 5. (G) Volcano plot showing DEGs in cluster 7. (H) GO enrichment analysis of DEGs in cluster 7. (I) KEGG enrichment analysis of cluster 7.

There were 31 genes in cluster 2 (*TNFSF10+*) that differed between the two groups (Figure 5A). HBV‐infected pregnant women enriched in inflammatory activation pathways, such as cytokine activity, neutrophil chemotaxis, neutrophil degranulation and cytokine‐mediated signalling pathways. Of particular note, anti‐viral pathways were defective, such as the negative regulation of virus entry into host cells, negative regulation of virus genome replication, response to interferons and defensive response to the virus (Figure 5B). In addition, a KEGG pathway analysis suggested that HBV‐infected pregnant women enriched in Th17 cell differentiation, NF‐κB signalling pathway, and cytokine–cytokine receptor interactions, with a decrease in genes in the apoptosis pathway (Figure 5C). The proportion of this monocyte population was significantly lower in HBV‐infected pregnant women than in the healthy group. The increased inflammatory response and decreased antiviral activity as well as the anti‐apoptotic phenotype of monocytes suggested that cluster 2 plays an important role in the immune response against HBV, and an increase in this group of cells may prevent mother‐to‐child HBV intrauterine infection.

Cluster 5 (*MT1G+*) had a number of DEGs between HBV‐infected pregnant women and healthy pregnant women, including 33 upregulated genes and 128 downregulated genes (Figure 5D). Upregulated genes were involved in cell migration and the regulation of RNA splicing and transcription, and downregulated genes were involved in the inhibition of viral replication, interferon response, and antigen processing and presentation (Figure 5E). RNA splicing, is critical in eukaryotic gene regulation and plays an important role in maintaining T cell and B cell homeostasis in the peripheral immune system in normal pregnant women. A KEGG analysis showed enrichment for mineral absorption and a decrease in the PI3K pathway (Figure 5F). The frequency of cluster 5 was significantly higher in HBV‐infected pregnant women than in healthy controls. We hypothesized its antiviral activity was decreased by increasing mRNA splicing pathway.

Additionally, 72 genes were identified in cluster 7 (*TUBB1+*), including 58 upregulated genes and 14 downregulated genes in HBV‐infected pregnant women compared with healthy pregnant women (Figure 5G). Greater enrichment was observed in the negative regulation of growth, detoxification and response of metal ions, and inflammatory pathways, including cytokine activity, neutrophil chemotaxis and chemokine‐mediated signalling pathways when compared with profiles in healthy pregnant women. Similar to cluster 2, cluster 7 was also characterized by the down‐regulation of the antiviral response pathway (Figure 5H). However, different from cluster 2, the frequency of cluster 7 was elevated in HBV‐infected pregnant women, and the neutrophil degranulation pathway was downregulated. A KEGG analysis showed high enrichment for immune system‐related pathways, such as the TNF signalling pathway, Th17 cell differentiation, Th1 and Th2 cell differentiation, IL‐17 signalling pathway, toll‐like receptor signalling pathway, NF‐κB signalling pathway, chemokine signalling pathway and cytokine−cytokine receptor interactions, consistent with the GO analysis (Figure 5I).

## 4 DISCUSSION

Pregnancy and HBV infection are all accompanied by significant systemic immunological adaptations.^25^, ^26^ However, previous studies have primarily focused on innate immunity alteration of non pregnant patients with chronic hepatitis B, making it difficult to obtain comprehensive scenarios of cellular and molecular immune responses for HBV‐infected women. To address this issue, we profiled the immune landscape in PBMCs from HBV‐infected women at single‐cell resolution and determined the characteristics of cellular responses in HBV‐infected pregnant women. The results of this study showed that DEGs in PBMCs between HBV‐infected and healthy pregnant women were highly expressed in monocytes. There are also less studies on monocytes of HBV‐infected pregnant women. Therefore, we focused on the frequency, functional alterations, and transcriptome characteristics of monocytes in HBV‐infected pregnant women.

We explored single‐cell RNA sequencing to classify monocytes into eight clusters and two cell subtypes (classical and non‐classical monocytes). The proportion of monocytes increase during pregnancy beginning in the first trimester, meanwhile monocytes increase in chronic hepatitis B.^27^ In contrast, we observed that there was no significant difference in monocytes frequency between two groups, indicating pregnancy effects immune status of chronic hepatitis B in some extent. Moreover, the proportion of CM and NCM in PBMCs of HBV‐infected pregnant women did not significant change, and the ratio in monocytes did not differ between groups, indicating there was no transformation between the two subtypes, which might both contribute to the immune response to HBV infection in pregnant women.

An analysis of DEGs between the two groups suggested that *IL1B* and *IER3* were highly expressed only in monocytes, while metallothionein gene expression levels were relatively higher in monocytes. Thus, we propose monocytes play an important role in HBV‐infected pregnant women with a high viral load. *IL1B* encodes the cytokine IL‐1β, which is produced by monocytes in response to infection and is closely related to the innate immune response and immune regulation.^28^ After HBV infection, monocytes are activated and secrete the inflammatory mediator IL‐1β, enhancing the inflammatory response. The mRNA and protein levels of IL‐1β were higher in patients with chronic hepatitis B than in healthy controls, and IL‐1β levels were positively correlated with disease severity.^29^ Our results were consistent with previous results, and we also verified that *IL1B* mRNA and protein levels were increased in HBV‐infected pregnant women with a high viral load in vitro. Proteins encoded by *IER3* are stimulated by multiple stressors, including viral infection and IL‐1β, and play a complex role in cell cycle regulation and apoptosis for multiple cell types.^30^, ^31^, ^32^ HBV‐infected pregnant women have high expression of *IER3*, which may inhibit monocyte apoptosis during the immune response. Further studies are needed to evaluate the interaction between HBV infection, IL‐1β and *IER3*. Metallothionein genes not only participate in the detoxification of heavy metals, regulation of zinc homeostasis and scavenging of free radicals, but also play important roles in immune regulation and liver diseases.^33^, ^34^ The expression level of MT is correlated with disease progression in chronic hepatitis B^35^ with augment in early hepatitis B and decrease in advanced hepatitis B, cirrhosis and liver cancer. The elevated expression levels of *MT1* and *MT2* suggested that these genes may be related to immune activation and innate responses of monocytes to HBV infection. Compared with other white blood cells, monocytes showed the highest MT expression at the protein and mRNA levels and the ability to bind to metal zinc. The anti‐apoptotic phenotype of monocytes is associated with an elevation in MT in HIV.^36^ The results of our study showed that metallothionein was expressed at relatively high levels in monocytes of HBV‐infected pregnant women, including high expression levels in almost every monocyte cluster. The expression levels of *MT1X, MT2A* and *MT1E* were also increased in the monocyte line THP‐1 stimulated by serum of HBV‐infected pregnant women. We speculated that monocytes may induce MT gene expression during persistent HBV infection, and MT overexpression in monocytes may be involved in resistance to apoptosis.

Compared with healthy pregnant women, a function enrichment analysis showed that monocytes show immune response defects, as evidenced by a decreased ability to respond to interferon. In healthy pregnancy, monocytes exhibits anti‐inflammatory activity.^37^ On the contrary, our results showed many genes related to ‘cytokine activity’, ‘neutrophil chemotaxis’ and ‘inflammation response’ were upregulated in monocytes of HBV‐infected women, such as *IL1B, PTGS2, PPBP, CCL4* and so on. Each monocytes cluster of monocytes had unique gene expression characteristics and functional changes. *TNFSF10+* monocytes and *MT1G+* monocytes of HBV‐infected pregnant women showed an enhanced inflammatory response activity, promoting the defence against chronic HBV infection, while *TNFSF10+* monocytes, *MT1G+* monocytes and *TUBB1+* monocytes showed decreased antiviral activity, such as response to IFN, negative regulation of viral genome replication. Alternative RNA splicing via spliceosome pathway plays an important role in maintaining T cell and B cell homeostasis in the peripheral immune system.^38^ Upregulation of alternative splicing may contribute to the inhibition of B cell and T cell activity during pregnancy.^26^ In this study, *TUBB1+* monocytes enriched mRNA splicing pathway. We hypothesized that mRNA splicing pathway may play a part role in inhibition of monocytes. Hence, restoring the biological functions of these three clusters is a potential strategy to enhance the antiviral capacity in HBV‐infected pregnant women and may reduce the risk of vertical transmission of HBV from mother to child.

There are some limitations in this study. On one hand, it is necessary to augment the sample size, which can better support our research results. On the other hand, this study is mostly descriptive analysis, which shows alteration of transcription profiles and biological processes of monocytes in HBV‐infected pregnant women. Data mining of single‐cell RNA sequencing is insufficient, and further research on the mechanism of innate immune response is still needed. In the next step, the specific mechanism will be verified in monocytes of HBV‐infected pregnant women by experimental methods based on differential expressed genes and molecular pathways found in this study, in order to deepen the understanding of the immune changes of HBV‐infected pregnant women. The interaction between monocytes and other cells in PBMC will also be the direction of our further research.

In summary, our study reveals the transcriptional profiles of monocytes in HBV‐infected pregnant women during the middle trimester of pregnancy and expands our understanding of the alterations of monocytes subsets. Furthermore, we will deeply explore interaction between monocyte clusters and other immune cells to uncover underlying mechanism of monocytes in HBV pregnant women.

## AUTHOR CONTRIBUTIONS

**Hongyan Wang:** Formal analysis (lead); validation (lead); visualization (lead); writing – original draft (lead). **Xia Li:** Methodology (equal); validation (equal). **Kaiyue Yang:** Methodology (supporting); validation (supporting). **Fanfan Guo:** Supervision (equal); validation (equal). **Xiaona Wang:** Validation (supporting). **Ziyan Zhao:** Validation (supporting). **Yanju Jia:** Validation (supporting). **Fan Gao:** Formal analysis (equal); methodology (supporting); supervision (supporting). **Guiqin Bai:** Funding acquisition (lead); investigation (equal); methodology (equal); project administration (lead).

## CONFLICT OF INTEREST STATEMENT

The authors declare no competing interests.

## Supporting information


**TABLE S1** Clinical and virological parameters.
**TABLE S2** Primer sequences.
**FIGURE S1** DEGs in monocyte subclusters 1, 3, 4, 6 and 8 and functional enrichment analyses. (A) Volcano plot showing the differentially expressed genes in cluster 1 between HBV‐infected pregnant women and healthy pregnant women. (B) GO enrichment analysis of DEGs in cluster 1 between CASE and CON. (C) Volcano plot showing DEGs in cluster 3. (D) GO enrichment analysis of DEGs in cluster 3. (E) Volcano plot showing DEGs in cluster 4. (F) GO enrichment analysis of DEGs in cluster 4. (G) Volcano plot showing DEGs in cluster 6. (H) GO enrichment analysis of DEGs in cluster 6. (I) Volcano plot showing DEGs in cluster 8. (J) GO enrichment analysis of DEGs in cluster 8.

## Data Availability

The single‐cell RNA sequencing data generated in this study can be found in the SRA database [SRA accession: PRJNA645249].
