# Supplementary material for: Single‐cell RNA sequencing reveals transcriptional profiles of monocytes in HBV‐infected pregnant women during mid‐pregnancy
Source: J Cell Mol Med. 2023 Apr 20;27(11):1465–76. doi: 10.1111/jcmm.17746 (PMC10243157; doi:10.1111/jcmm.17746)
Supplement: Supplementary file 1 — TABLE S1 Clinical and virological parameters. TABLE S2 Primer sequences. FIGURE S1 DEGs in monocyte subclusters 1, 3, 4, 6 and 8 and functional enrichment analyses. (A) Volcano plot showing the differentially expressed genes in cluster 1 between HBV‐infected pregnant women and healthy pregnant women. (B) GO enrichment analysis of DEGs in cluster 1 between CASE and CON. (C) Volcano plot showing DEGs in cluster 3. (D) GO enrichment analysis of DEGs in cluster 3. (E) Volcano plot showing DEGs in cluster 4. (F) GO enrichment analysis of DEGs in cluster 4. (G) Volcano plot showing DEGs in cluster 6. (H) GO enrichment analysis of DEGs in cluster 6. (I) Volcano plot showing DEGs in cluster 8. (J) GO enrichment analysis of DEGs in cluster 8. [file JCMM-27-1465-s001.docx]

Supplementary Table 1 Clinical and virological parameters

|  | Gestational  weeks | Age  (years old) | HBsAg  (IU/mL) | Anti-HBs (mIU/mL) | HBeAg  (COI) | Anti-HBe  (COI) | Anti-HBc  (COI) | AST  (U/L) | ALT  (U/L) | HBV DNA  (IU/mL) |
| --- | --- | --- | --- | --- | --- | --- | --- | --- | --- | --- |
| CON1 | 24^+1^ | 25 | - | - | - | - | - | 20 | 23 | none |
| CON2 | 24^+6^ | 32 | - | 224.54 | - | - | - | 16 | 10 | none |
| CON3 | 25^+0^ | 30 | - | 354.29 | - | - | - | 23 | 17 | none |
| HBV1 | 22^+6^ | 33 | 73890.16 | 0.26 | 1474.534 | 61.11 | 8.59 | 13 | 13 | 1.31×10^8^ |
| HBV2 | 22^+4^ | 26 | 32301.07 | 0.06 | 1288.78 | 56.16 | 10.35 | 19 | 16 | 8.75×10^7^ |
| HBV3 | 22^+2^ | 27 | 33790.09 | 0.82 | 1030.806 | 55.51 | 6.57 | 17 | 15 | 4.23×10^7^ |
| Reference range |  |  | <0.05 | <10 | <1.00 | >1.00 | <1.00 | 13-35 | 7-40 | <1.00×10^2^ |

Supplementary Table 2 Primer sequences

| HLA-DQB1 | Forward | GCGGGATCTTGCAGAGGAG |
| --- | --- | --- |
|  | Reserve | ACTTTGATCTGGCCTGGATAGAA |
| RPS10 | Forward | ATGTTGATGCCTAAGAAGAACCG |
|  | Reserve | CGTAGCCTCGGGACTTGAGA |
| IER3 | Forward | CAGCCGCAGGGTTCTCTAC |
|  | Reserve | GATCTGGCAGAAGACGATGGT |
| IL-1B | Forward | ATGATGGCTTATTACAGTGGCAA |
|  | Reserve | GTCGGAGATTCGTAGCTGGA |
| Metallothionein1X | Forward | AACTCCTGCTTCTCCTTGCC |
|  | Reserve | GCTCTATTTACATCTGAGAGCACAA |
| Metallothionein2A | Forward | AACCTGTCCCGACTCTAGC |
|  | Reserve | GGAATATAGCAAACGGTCACG |
| Metallothionein1E | Forward | TCAGGTTGGGAGGGAACTCAA |
|  | Reserve | GAAAGCCTGGAGAGGGAATGA |
| MYOM2 | Forward | GAGAGACACACATTTGAAGAGCG |
|  | Reserve | TCCTGTACTTTCCCGGTTCAG |
| MT1F | Forward | TGGTTCCTGCAAGTGCAAAGAGTG |
|  | Reserve | GGAATGTAGCAAATGGGTCAAGGTGG |


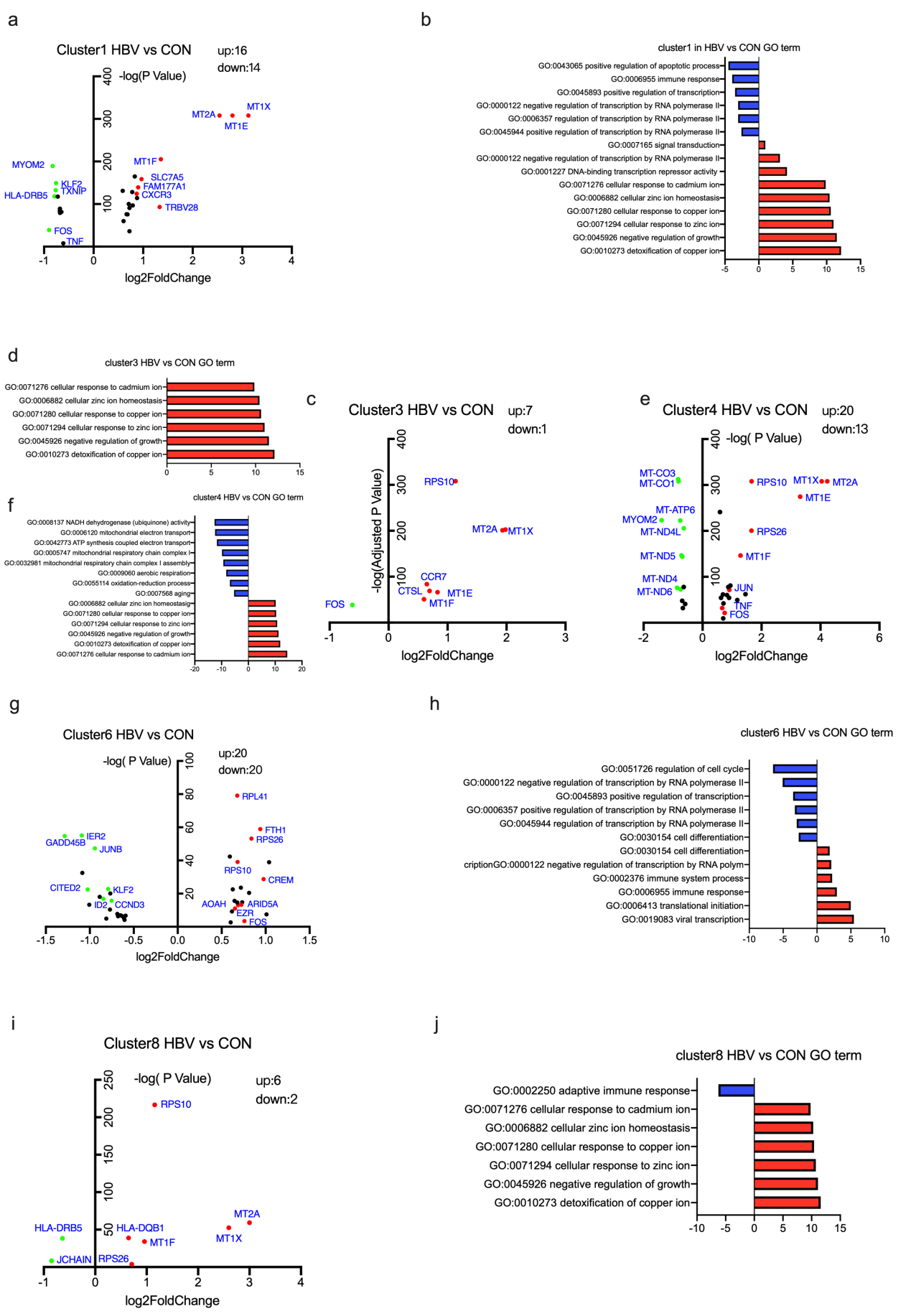


Supplementary figure1 . DEGs in monocyte subclusters 1,3,4,6 and 8 and functional enrichment analyses.

**a** Volcano plot showing the differentially expressed genes in cluster 1 between HBV-infected pregnant women and healthy pregnant women.

**b** GO enrichment analysis of DEGs in cluster 1 between CASE and CON.

**c** Volcano plot showing DEGs in cluster 3.

**d** GO enrichment analysis of DEGs in cluster 3.

**e** Volcano plot showing DEGs in cluster 4.

**f** GO enrichment analysis of DEGs in cluster 4.

**g** Volcano plot showing DEGs in cluster 6.

**h** GO enrichment analysis of DEGs in cluster 6.

**i** Volcano plot showing DEGs in cluster 8.

**j** GO enrichment analysis of DEGs in cluster 8.
